# BAFF blockade attenuates acute graft-versus-host disease directly *via* the dual regulation of T- and B-cell homeostasis

**DOI:** 10.3389/fimmu.2022.995149

**Published:** 2022-12-06

**Authors:** Youngwoo Jeon, Jung-Yeon Lim, Keon-Il Im, Nayoun Kim, Seok-Goo Cho

**Affiliations:** ^1^ Department of Hematology, Yeouido St. Mary's Hospital, College of Medicine, The Catholic University of Korea, Seoul, South Korea; ^2^ Lymphoma and Cell Therapy-Research Center, Yeouido St. Mary Hospital, College of Medicine, The Catholic University of Korea, Seoul, South Korea; ^3^ Institute for Translational Research and Molecular Imaging, The Catholic University of Korea, Seoul, South Korea; ^4^ Department of Biomedical Laboratory Science, Inje University, Kimhae, South Korea; ^5^ Department of Hematology, Seoul St. Mary's Hematology Hospital, College of Medicine, The Catholic University of Korea, Seoul, South Korea

**Keywords:** graft-versus-host disease, hematopoietic stem cell transplantation, B-cell-activating factor (BAFF), belimumab, regulatory T-lymphocytes

## Abstract

**Introduction:**

B-cell-activating factor (BAFF) is associated with donor-specific antibodies and chronic graft-versus-host disease (GVHD) after allogeneic hematopoietic stem cell transplantation (allo-HSCT). However, the effects of BAFF on T-cell physiological function have not been fully elucidated in acute GVHD.

**Methods:**

We examined the effects of belimumab, a monoclonal antibody targeting BAFF, for the treatment of acute GVHD. We examined the effects of T cells and B cells separately when inducing GVHD in mouse model.

**Results:**

Therapeutic functional manipulation of endogenous BAFF can improve acute GVHD during the early post-transplant period. In this study, BAFF was shown to increase the proportions of CD4^+^IL-17^+^, CD4^+^IL-6^+^ Th17, and CD4^+^IFN-γ^+^ Th1 cells and to reduce the proportion of regulatory T (Treg) cells. Furthermore, the belimumab therapy group showed increased B220^+^IgD^+^IgM^+^ mature B cells but decreased B220^+^IgD^−^IgM^−^ memory B cells, B220^+^Fas^+^GL-7^+^ germinal center formation, and B220^+^IgD^−^CD138^+^ plasma cells. These results indicate that BAFF can alleviate acute GVHD by simultaneously regulating T and B cells. Interestingly, the BAFF level was higher in patients with acute GVHD after HSCT compared with patients receiving chemotherapy.

**Conclusion:**

This study suggests that BAFF blockade might modulate CD4 +T-cell-induced acute GVHD early after allo-HSCT and the possibility of simultaneously controlling chronic GVHD, which may appear later after allo-HSCT.

## Introduction

B-cell-activating factor (BAFF), a member of the tumor necrosis factor (TNF) family, is an important B-cell survival factor that is expressed primarily by monocytes, macrophages, dendritic cells, neutrophils, and mast cells and functions to stimulate the proliferation, differentiation, and survival of B cells (1). In addition, the maintenance of B-cell homeostasis is dependent on the concentration of soluble BAFF *in vivo* (2). A high level of BAFF is known to promote autoreactive B cells in autoimmune diseases such as systemic lupus erythematosus and Sjögren syndrome (3). Changes in the level of BAFF are related to alterations in B-cell homeostasis.

After allogeneic hematopoietic stem cell transplantation (allo-HSCT), immunological conditioning results in the recovery of naive B cells before B-cell homeostasis is reached (2). There is a direct correlation between the serum BAFF concentration and the severity of chronic graft-versus-host disease (GVHD) after allo-HSCT. Relative B lymphopenia and an elevated BAFF level after allo-HSCT may support pathological activated alloreactive and autoreactive B-cell populations in patients with chronic GVHD. In addition, earlier studies had indicated a role of B cells in GVHD in experimental studies and in a patient’s blood sample: the experimental work by R. Renkonen and P. Hayr showed an increased proportion of B cells in the target organs of acute GVHD (4); also significant increased numbers of immunoglobulin-expressing B cells were found in target organs during acute GVHD by Dariusz et al. (5); there was also an increased *in-vitro* B-cell IgG production during acute GVHD in another study (6); and another clinical study showed that patients treated with rituximab prior to hematopoietic stem cell transplantation, for instance for B-cell lymphoma, had decreased probability of acute GVHD compared with patients not given anti-B-cell antibodies (7).

The BAFF/BAFF-receptor (BAFF-R) pathway is important for T-cell activation, proliferation, and differentiation (8). Specifically, host responses to transplantation can significantly reduce the therapeutic effects by targeting the binding of BAFF to BAFF-R expressed by T cells (9). In autoimmune diseases, the proinflammatory cytokines interferon (IFN)-γ and TNF-β can induce BAFF expression, which may inhibit apoptosis of B cells in inflammatory microenvironments and increase autoantibody production *in vivo* (10). Constitutive overexpression of BAFF promotes Th17 cell generation *in vitro* and *in vivo* and aggravates the manifestation of Th17 cell-driven autoimmune disease (11).

Although acute GVHD after allo-HSCT has classically been assumed to be a Th1-mediated response based on findings in animal models as well as clinical data, differentiated Th17 cells were shown to mediate severe acute GVHD *in vitro* (12, 13).

This study examined a means of regulating GVHD by blocking its effect. Our results suggest that it is possible to prevent acute GVHD by targeting Th1 and Th17 cells by blocking BAFF signaling with belimumab during the early post-transplant period.

## Methods

### Mice

C57BL/6 (H-2b) and BALB/c (H-2d) mice, 8–10 weeks old, were purchased from Orient Bio (Seongnam, Korea). The mice were kept under specific pathogen-free conditions in an animal facility with controlled humidity (55% ± 5%), light (12/12 h light/dark), and temperature (22°C ± 1°C). The air in the facility was passed through a HEPA filter system designed to exclude bacteria and viruses. The animals were fed mouse chow and tap water *ad libitum.* This animal experiment was approved by the Institutional Animal Care and Use Committee of the School of Medicine, Catholic University of Korea, in accordance with the Laboratory Animals Welfare Act (approval number: CUMC-2015-0097-02). The protocols used in this study were approved by the Animal Care and Use Committee of the Catholic University of Korea.

### Bone marrow transplantation and induction of acute GVHD

As BAFF affects both T cells and B cells, we examined the effects of T cells and B cells separately when inducing GVHD in our mouse model. GVHD protocol 1 consisted of the irradiation of recipient mice (BALB/c, H-2d) at a dose of 800 cGy followed by the intravenous (IV) injection of 5 × 10^6^ bone marrow cells and 5 × 10^6^ spleen cells from donor mice (C57BL, H-2b). Mice in the control group were irradiated at the same dose but received only 5 × 10^6^ bone marrow cells from donor mice (C57BL/6, H-2b), which did not induce GVHD. GVHD protocol 2 consisted of the irradiation of recipient mice (BALB/c, H-2d) at a dose of 800 cGy followed by the IV injection of 5 × 10^6^ T-cell-depleted bone marrow (TCD-BM) cells and 2.5 × 10^6^ CD4^+^ splenic T cells from donor mice (C57BL/6, H-2b). Mice in the control group were irradiated at the same dose but received only 5 × 10^6^ TCD-BM cells from donor mice (C57BL/6, H-2b), which did not induce GVHD. Survival after bone marrow transplantation (BMT) was monitored daily, and the degree of clinical GVHD was assessed weekly by scoring changes in five clinical parameters: weight loss, posture, activity, fur texture, and skin integrity.

### Belimumab treatment

Mice were injected intraperitoneally (IP) with 200 μg of belimumab (Benlysta^®^; GlaxoSmithKline, London, UK) twice a week after BMT (BMT + days 0, 4). Control mice received IP injections of 200 μg of human IgG1 antibodies at the same time points (14).

### Flow cytometry

Mononuclear cells were immunostained with various combinations of the following fluorescence-conjugated antibodies: CD4 (eBioscience, San Diego, CA, USA), CD25 (eBioscience), Foxp3 (eBioscience), IFN-γ (eBioscience), interleukin (IL)-4 (BD Pharmingen, San Diego, CA, USA), IL-17 (eBioscience), B220 (BioLegend, San Diego, CA, USA), IgD (eBioscience), IgM biotin (BD Pharmingen), CD138 (BD Pharmingen), CD23 (eBioscience), CD21 Biotin (eBioscience), Fas (CD95) (BD Pharmingen), and streptavidin (BD Pharmingen). Before intracellular cytokine staining, the cells were stimulated in a culture medium containing phorbol myristate acetate (25 ng/ml; Sigma-Aldrich, St. Louis, MO, USA), ionomycin (250 ng/ml; Sigma-Aldrich), or monensin (GolgiStop, 1 μl/ml; BD Pharmingen) in an incubator under an atmosphere of 5% CO_2_ at 37°C for 4 h. Intracellular staining was performed using an intracellular staining kit (eBioscience) according to the manufacturer’s protocol. Flow cytometric analysis was performed on a FACS LSRFortessa instrument (BD Biosciences, Franklin Lakes, NJ, USA).

### Enzyme-linked immunosorbent assay

Serum BAFF concentrations were measured by sandwich enzyme-linked immunosorbent assays (ELISAs) as follows: anti-mouse/human BAFF monoclonal antibodies (R&D Systems, Minneapolis, MN, USA) were added to 96-well plates (Nunc, Roskilde, Denmark) and incubated overnight at 4°C. The wells were blocked with blocking solution [PBS containing 1% bovine serum albumin (BSA) and 0.05% Tween 20] for 2 h at room temperature. Test samples and standard recombinant mouse/human BAFF (R&D Systems) were added to separate wells, and the plates were incubated at room temperature for 2 h, after which they were washed. Biotinylated BAFF polyclonal antibodies (R&D Systems) were added, and the reaction was allowed to proceed for 2 h at room temperature. The plates were washed, ExtrAvidin-alkaline phosphatase (1:2,000 dilution; Sigma-Aldrich) was added, and the reaction was allowed to proceed for an additional 2 h. The plates were washed and 50 μl of *p*-nitrophenyl phosphate disodium salt (Pierce Chemical Co., Rockford, IL, USA) diluted to 1 mg/ml in diethanolamine buffer was applied. The experiments were carried out in accordance with the manufacturer’s instructions.

### Isolation of splenocytes and CD4^+^ T cells

The isolation of mouse splenocytes and splenic CD4^+^ T cells and the differentiation of effector T cells were performed as described previously (15). To purify splenic CD4^+^ T cells, mouse splenocytes were incubated with CD4-coated magnetic beads and isolated using magnetic-activated cell sorting separation columns (Miltenyi Biotech, Auburn, CA, USA). To establish Th17 cell polarization conditions, sorted CD4^+^ T cells were stimulated with plate-bound anti-CD3 (1 μg/ml), anti-CD28 (1 μg/ml), anti-IFN-γ (2 μg/ml), anti-IL-4 (2 μg/ml), TGF-β (2 ng/ml), and IL-6 (20 ng/ml) for 72 h. Each experiment was conducted using 10 mice and repeated several times, the survival graph was created, and *ex-vivo* experiments on mice were performed in different batches.

For Th17 cell differentiation, splenic CD4^+^ T cells from normal C57BL/6 mice were stimulated with anti-CD3/anti-CD28/anti-IFN-γ/anti-IL-4/recombinant TGF-β/recombinant IL-6 monoclonal antibodies with or without recombinant BAFF for 72 h. For the allogeneic response, splenic CD4^+^ T cells from normal C57BL/6 mice were stimulated with splenic non-CD4^+^ T cells from BALB/c mice with or without recombinant BAFF for 72 h. For Th0 cell differentiation, splenic CD4^+^ T cells from normal C57BL/6 mice were stimulated with anti-CD3/anti-CD28 antibodies for 72 h. The cells were then stained with anti-CD4, anti-BAFF-R, anti-IL-17, anti-IL-6, anti-CD25, anti-Foxp3, anti-IFN-γ, or anti-IL-4 antibodies for flow cytometry on BMT + 17 days.

### Evaluation for histopathology of acute GVHD

Mice were sacrificed 17 days after BMT for blinded histopathological analysis of aGVHD targets (liver and small intestine). Organs were harvested, cryoembedded, and then sectioned. Tissue sections were fixed in 10% buffered formalin (Sigma-Aldrich) and stained with hematoxylin (Sigma-Aldrich) and eosin Y 1% solution (Muto Pure Chemical Co., Ltd., Tokyo, Japan) for histological examination. The scoring system for each parameter was 0 for normal, 0.5 for focal and rare, 1 for focal and mild, 2 for diffuse and mild, 3 for diffuse and moderate, and 4 for diffuse and severe, in accordance with previously reported aGVHD histology.

### Statistical analysis

Statistical analyses were performed using GraphPad Prism (ver. 5.01) and SAS version 9.2 (SAS Institute, Inc., Cary, NC, USA), and all statistical tests were two-sided. *p*-values <0.05 were considered statistically significant. Comparisons between groups (three or more groups) were analyzed statistically using the Kruskal–Wallis with Dunn’s post-test. Pairwise group comparisons used the Mann–Whitney *U* test, and *p*-values were adjusted for multiple comparisons using Bonferroni’s method to determine the statistical significance of these comparisons. Survival analyses were performed with the Kaplan–Meier and log-rank (Mantel–Cox) method of survival distribution. In all analyses, *p*-values less than 0.05 were considered to indicate statistical significance.

## Results

### BAFF modulates Th1, Th17, and Treg differentiation of donor CD4^+^ T cells *in vitro*

**Figure 1:**
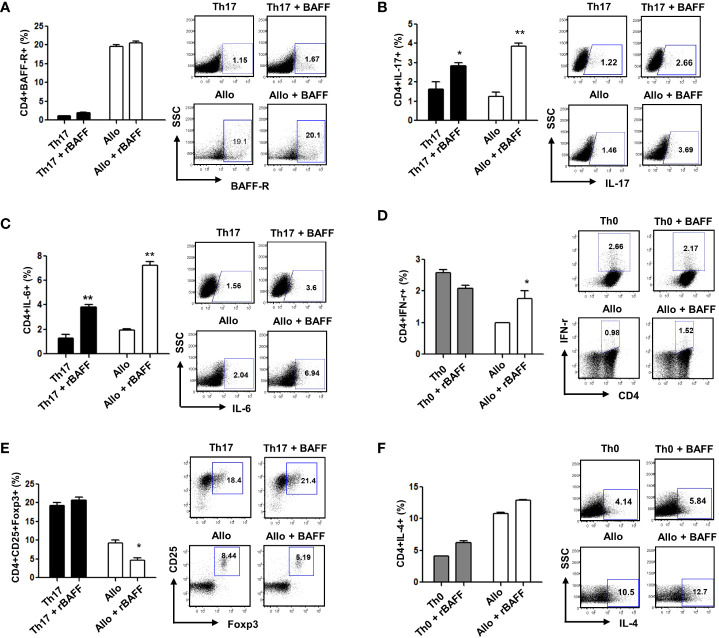
To evaluate GVHD and treatment response in each group, immune cells were examined on the 17th day after bone marrow transplantation. **(A)** There was no difference in BAFF-R expression according to the presence or absence of recombinant BAFF (rBAFF). BAFF promoted Th1 and Th17 cell differentiation and suppressed Treg cell differentiation in vitro; The levels of **(B)** CD+IL-17, **(C)** CD+IL-6, and **(D)** CD4+IFN-γ expression increased, while **(E)** CD4+CD25+Foxp3+Treg cells decreased in recombinant BAFF-treated CD4+ T cells compared to untreated control CD4+ T cells. **(F)** The expression of BAFF-R and IL-4 did not differ between BAFF-treated and untreated control CD4+ T cells. Representative results are shown. * p < 0.05; ** p < 0.01 (Student’s t test).

BAFF is related to the induction and maintenance of T- and B-cell homeostasis. BAFF was shown to promote T-cell activation with cytokine production *via* BAFF-R *in vitro* and *in vivo* (8). To evaluate GVHD and treatment response in each group, immune cells were examined on the 17th day after bone marrow transplantation. Our results showed that BAFF-R expression on CD4^+^ T cells was higher in the allogeneic response than in the Th17 inflammatory environment, but there was no difference in BAFF-R expression according to the presence or absence of recombinant BAFF (rBAFF) (**Figure 1A**). In the Th17 inflammatory environment and allogeneic response, we confirmed that CD4^+^IL-17^+^ and CD4^+^IL-6^+^ Th17 cells and CD4^+^IFN-γ^+^ T-cells were increased by rBAFF (**Figures 1B** **–D**). However, CD4^+^CD25^+^Foxp3^+^ Treg cells were decreased by rBAFF in the allogeneic response (**Figure 1E**). In addition, there were no differences in Th2 cells according to the presence or absence of rBAFF (**Figure 1F**). These results indicated that BAFF promotes effector T-cell differentiation with cytokine production in allogeneic responses.

### Blocking of BAFF minimally attenuates acute GVHD after allo-BMT with whole spleen cells

**Figure 2:**
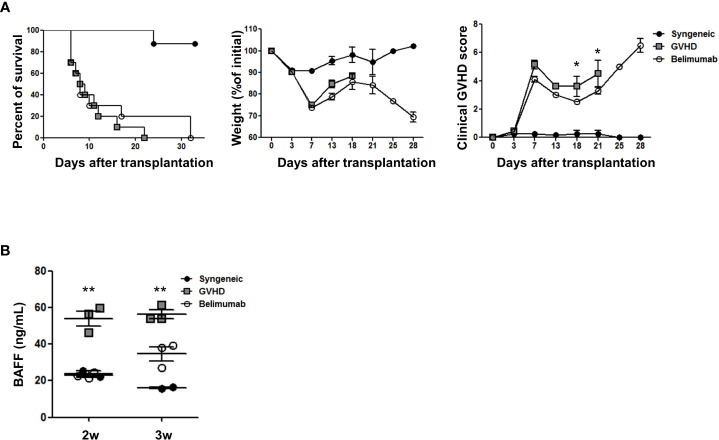
Blocking of BAFF signaling mitigated acute GVHD-induced mortality and target organ injury in an experiment using GVHD protocol 1. Bone marrow transplantation (BMT) and acute GVHD induction were performed as described in the *Methods* (GVHD protocol 1). **(A)** The survival, weight, and clinical GVHD score of the animals were monitored. Survival was not improved using anti-BAFF compared with the acute GVHD experimental mice and was worse compared with the syngeneic controls. However, GVHD scores were improved compared with GVHD experimental mice, and there was no significant difference in weight between the two groups (*n* = 8 mice/group). **(B)** The belimumab group showed lower levels of BAFF in serum 2 and 3 weeks after acute GVHD induction on ELISA. *p < 0.05; ** p < 0.01 (comparisons between the three groups were analyzed statistically using the Kruskal–Wallis with Dunn’s post-test).

To evaluate BAFF blockade *in vivo*, we established an acute GVHD mouse model (GVHD protocol 1) with irradiation at a dose of 800 cGy and IV injection of 5 × 10^6^ bone marrow cells and 5 × 10^6^ spleen cells from donor mice (C57BL/6, H-2Kb) as a basic GVHD research. On days 0 and 4 after the induction of GVHD, mice were administered 200 μg of belimumab to block BAFF production. Mice were monitored at different time points after the induction of GVHD for survival, body weight, and clinical GVHD score. To determine changes in T and B cells, recipient mice were transplanted with whole donor bone marrow and spleen cells. In this model, survival was not improved using anti-BAFF compared with the acute GVHD experimental mice in the early stage of BMT and was worse compared with the syngeneic controls. However, the survival rate showed a tendency to improve in the late stage of BMT of the anti-BAFF group. Also, GVHD scores were improved compared with GVHD experimental mice after BMT (**Figure 2A**). Interestingly, BAFF levels were significantly increased in the acute GVHD mouse model in the early period after BMT (**Figure 2B**). These results showed that the BAFF level is related to chronic GVHD (16, 17) and that it plays an important role in the acute GVHD model, too.

### Blocking of BAFF inhibits acute GVHD through reciprocal regulation of Th1/Th2 and Th17/Treg cells after BMT + 17 days

**Figure 3:**
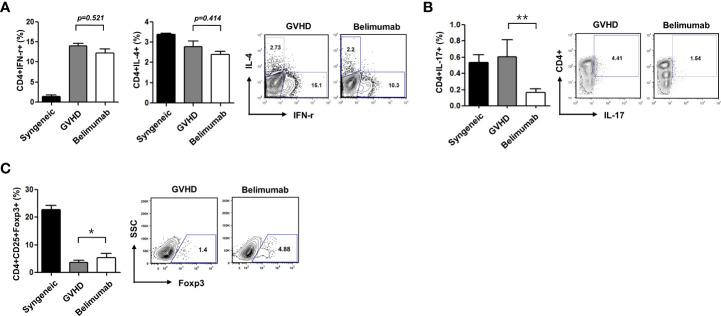
Blocking of BAFF attenuated acute GVHD through reciprocal regulation of Th1/Th2 and Th17/Treg cells. Intracellular cytokine profiles of splenic CD4^+^ cells 17 days after allo-BMT. Gated CD4^+^ T cells are shown in spleen cells. **(A)** The expression of CD4^+^IFN-r^+^ was not different between the GVHD group and the belimumab group (p = 0.521). The expression of CD4^+^IL-4^+^ was not statistically different between the two groups either (p = 0.414). **(B)** the expression of CD4^+^IL-17^+^ was decreased in the belimumab group compared with the recipients of the GVHD experiment (*p* = 0.008). **(C)** Also, the CD4^+^CD25^+^Foxp3^+^ cell population was increased in the belimumab-treated group (*p* = 0.042). *p < 0.05; ** *p* < 0.01 (comparisons between the three groups were analyzed statistically using the Kruskal–Wallis with Dunn’s post-test).

BAFF promotes BAFF-R expression in T lymphocytes (18) and contributes to T-cell activation (19). To confirm the immunoregulatory functions of a BAFF blockade related to Th1, Th2, Th17, and Treg cells, we measured the expression of cytokines and transcription factors in recipient mice. Flow cytometry showed that CD4^+^IFN-γ^+^ (*p* = 0.521) and CD4^+^IL-4^+^ (*p* = 0.414) were not statistically different between the GVHD group and the belimumab-treated group, then belimumab treated group had shown a tendency to decrease populations of CD4^+^IFN-γ^+^ and CD4^+^IL-4 (**Figure 3A**). CD4^+^IL-17^+^ cells were also decreased by belimumab therapy (**Figure 3B**) with statistical difference (*p* = 0.008). Moreover, there was an increased CD4^+^CD25^+^Foxp3^+^ population (**Figure 3C**) compared with that in GVHD experimental recipients (*p* = 0.042). Similar to the *in-vitro* results, there was no statistical difference in CD4^+^IFN-γ^+^ between the two groups (*p* = 0.521), and CD4^+^IL-4^+^ populations also did not differ between the two groups statistically (*p* = 0.414). These observations showed that after the onset of disease, belimumab could modulate the immunoregulatory effects of effector T cells.

### Blocking of BAFF modulates B-cell homeostasis

**Figure 4:**
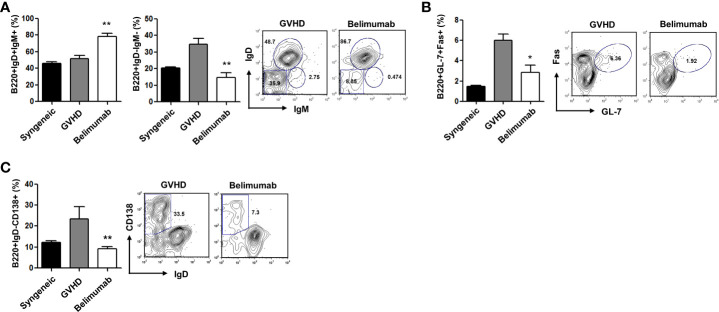
The effects of blocking BAFF were related to the differentiation of B-cell subsets. Distributions of B-cell phenotypes in the belimumab and the control groups after allo-BMT. **(A–C)** Low levels of B220^+^IgM^−^IgD^−^ memory B cells and B220^+^GL-7^+^Fas^+^ germinal center cells in the spleens of the belimumab group. B220^+^IgM^+^IgD^+^ mature B cells were present at a high frequency among gated B220^+^ splenocytes. Representative results are shown. *p < 0.05; **p < 0.01 (Kruskal–Wallis test method).

A high BAFF level promotes an altered B-cell compartment in the chronic GVHD mouse model (16). To determine the role of a BAFF blockade in the B-cell responses generated during T- and B-cell interactions, we carried out signaling experiments using different subsets of B cells. Based on the above observations and in agreement with the results of *ex-vivo* analyses, we found that the belimumab therapy group had an increased proportion of mature B cells (B220^+^IgM^+^IgD^+^), as well as a smaller proportion of immature B cells (B220^+^IgM^+^IgD^−^) and memory B cells (B220^+^IgM^−^IgD^−^), than the GVHD experimental group (**Figure 4A**). BAFF promotes the formation of germinal centers and plasma cells with induced immunoglobulins (20). We found that blocking BAFF signaling produced a lack of B220^+^GL-7^+^Fas^+^ germinal center cells (**Figure 4B**) and B220^+^CD138^+^IgD^−^ plasma cells (**Figure 4C**). Acute GVHD is likely to be reduced in the absence of T cells; these results may also indicate that changes in the subsets of B cells are independent of the severity of acute GVHD. Therefore, although B cells are not required for the development of acute GVHD, BAFF directly regulates B cells which could potentially contribute to the immune pathology of acute GVHD. Early clinical trials of BAFF blocking with belimumab demonstrated beneficial effects on both acute and chronic GVHD, and these results suggest that elevated BAFF levels contribute to B-cell activation in patients with active chronic GVHD (17, 21).

### Blocking of BAFF attenuates acute GVHD after allo-BMT with only CD4^+^ T cells

**Figure 5:**
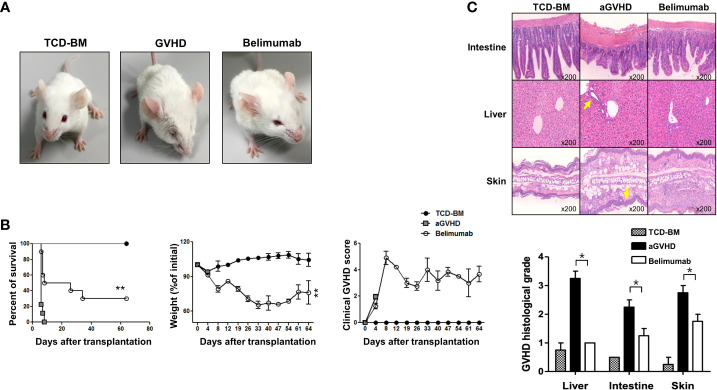
Blocking of BAFF signaling inhibited CD4^+^ T-cell-induced acute GVHD. BMT and the induction of acute GVHD were performed as described in the *Methods* (GVHD protocol 2). **(A)** Comparison of pathological skin lesions of acute GVHD model mice with belimumab treatment and controls on day 10 after transplantation (*n* = 8 mice/group). BALB/c recipients transplanted with bone marrow and spleen cells from wild-type mice showed chronic dermatitis on the ears, around the eyes, and on the back, and they developed alopecia. The belimumab group did not show dermatitis or hair loss. **(B)** The survival, weight, and clinical GVHD score of the animals were monitored. The belimumab group showed an average survival of 60 days. **p < 0.01 (Student’s *t*-test). **(C)** Histopathologic findings and scores in each group after allo-BMT (BMT + 17 days). Histologic scores were assessed in the liver, intestine, and skin. The belimumab therapy group showed markedly reduced lymphocyte infiltration and GVHD scores in intestinal tissue, liver, and subcutaneous skin lesion compared with the GVHD group. Statistical significance was determined by Student’s two‐tailed *t*‐test and ANOVA with Bonferroni correction for multiple comparisons; *p < 0.05.

In **Figure 2**, in the general GVHD induction experiment (GVHD protocol 1), there was no significant difference in the survival outcomes between the GVHD and the belimumab-treated groups. Therefore, to determine how BAFF directly affects T cells without affecting B cells or without interference effect on other immune cells (such as APC, interleukins, and so on), we examined whether belimumab could modulate acute GVHD by transplanting only splenic CD4^+^ T cells using GVHD protocol 2. Surprisingly, the recipients of belimumab therapy showed mild fur ruffling without scaling of the skin (**Figure 5A**). All recipients in the GVHD experimental group died early after BMT. In this model using GVHD protocol 2, all belimumab therapy recipients showed significantly longer survival, increased weight, and a markedly reduced clinical GVHD score compared with the GVHD experimental group (**Figure 5B**). These results confirmed that blocking BAFF signaling attenuated acute GVHD by regulating only CD4^+^ T and CD220^+^ B cells. All mice in the control group died by day +7 post-BMT. Based on the number of T cells added to the transplant, it is not something common to have GVHD mice dying as early as 1 week post-BMT. Therefore, when the histological findings of the organs of each experimental group were investigated on the same day in order to differentiate the death due to causes other than GVHD, it was confirmed that the death was due to GVHD (**Figure 5C**).

### Study of human serum whether BAFF plays a role in GVHD than systemic chemotherapy

Previous studies have shown that the BAFF/BAFF-R pathway plays a role in the development of GVHD and may represent a novel therapeutic target for the treatment or prevention of this disease (17). Previously, our research group reported that serum BAFF levels were significantly increased in patients with acute GVHD after allo-HSCT compared with patients without acute GVHD. Furthermore, it was necessary to confirm whether the serum BAFF level could be increased by chemotherapy alone or whether the increase in serum BAFF was due to the immune response of acute GVHD rather than the response to systemic chemotherapy. Therefore, we measured the serum BAFF concentration in healthy adults, in the group receiving systemic chemotherapy alone, and in the group developing acute GVHD after allo-HSCT. The analyzed patient groups are as follows: five of healthy donors, seven of patients in the high-intensive chemotherapy group who developed grade 3 neutropenia after systemic chemotherapy and T-cell lymphoblastic lymphoma and received more than 4 cycles of hyperCVAD/HDMTX-ara alternating with the chemotherapy regimen, and four acute GVHD patients who received allo-BMT after a third line or higher chemotherapy with refractory/relapsed diffuse large B-cell lymphoma.

**Figure 6:**
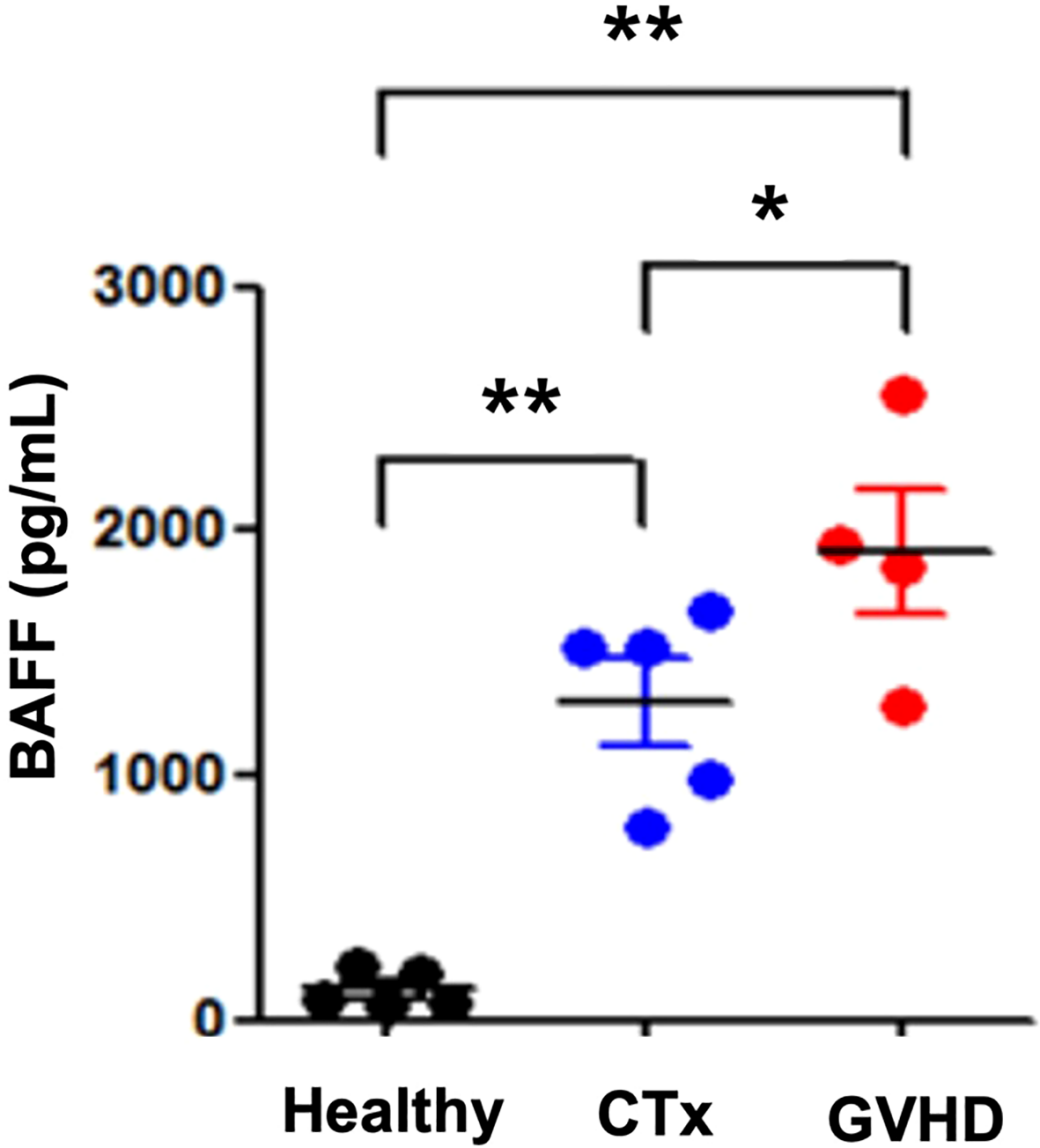
Serum BAFF levels in patients with GVHD at the first evaluation. An ELISA was performed to determine BAFF levels in serum samples from patients treated with chemotherapy, patients with GVHD, and healthy controls. Representative results are shown. GVHD patients had high levels of BAFF compared with patients receiving chemotherapy. *p < 0.05; **p < 0.01 (comparisons between the three groups were analyzed statistically using the Kruskal–Wallis with Dunn’s post-test).

In addition, to confirm the results obtained in the mouse GVHD experimental model in clinical practice, serum BAFF levels were evaluated in both patients treated with chemotherapy alone and with acute GVHD after allo-HSCT at our institution. To examine whether BAFF plays a role in GVHD, we compared serum BAFF levels between five patients with systemic high-dose chemotherapy alone and four patients with acute GVHD (overall grade III or IV) after allo-HSCT. The serum BAFF levels were higher in patients with acute GVHD compared with those with high-dose chemotherapy alone (**Figure 6**).

It indicates that treatment with belimumab, which suppresses the serum level of BAFF, may alleviate severe GVHD in patients with acute GVHD occurring after HSCT.

## Discussion

Previous studies focused primarily on the regulation of BAFF through the blockade of IL-21 in acute GVHD (9). In the present study, we demonstrated that BAFF can regulate T cells and B cells using belimumab, which blocks BAFF directly in the acute GVHD model. As belimumab binds to murine BAFF with a much lower affinity than to human BAFF (14), efficacy studies of belimumab in murine acute GVHD models were not tractable. Then, in the previously published data by Abigail Benitez et al., as the belimumab study for systemic lupus erythematosus, a human BAFF inhibitor was used in a mouse model experiment, and they reported successful and favorable results: *in vitro*, it was confirmed that Foxp3^+^ Treg cells were decreased by adding recombinant BAFF, and *in vivo*, the increase in Treg cells was increased when belimumab was added (22). Also, compared with the *in-vivo* experiment results using mouse anti-BAFF antibodies indirectly, clear results were obtained in our study.

BAFF contributes to B-cell homeostasis and function in chronic GVHD after HSCT (23, 24). Determining the role of high BAFF levels in relation to B-cell reconstitution and attainment of B-cell tolerance versus whether high BAFF is related to the promotion of autoreactive B cells will be critical if we are to understand the manifestations of chronic GVHD (25). Previous studies showed that BAFF overexpression induces marked expansion of activated B cells, particularly marginal zone B cells, as well as hypergammaglobulinemia, autoreactive antibody production, and immune complex deposition. Furthermore, BAFF promotes germinal center formation in patients with GVHD (20). Based on the previously reported association between excess BAFF and cGVHD, an elevated BAFF level promotes NOTCH2 expression in B cells and maintains SYK protein expression after B-cell receptor (BCR) activation. Corroborating a pathological role for BAFF, excess BAFF and alloantigen were shown to work together to promote a circulating BCR-responsive B-cell pool and alloantibody production in patients with chronic GVHD (16). However, in addition to its effect on B cells, a recent work has shown that BAFF can promote T-cell activation, proliferation, and differentiation through BAFF-R in T cells (8, 19, 26).

Dysregulation of Th1 or Th2 responses may contribute to the pathogenesis of autoimmune and allergic diseases (8). Th1 cells that secrete IFN-γ and/or TNF-α are involved in the induction of BAFF in neutrophils, fibroblast-like synoviocytes (FLS), monocytes, dendritic cells, and T cells (10, 27, 28). In addition, BAFF overexpression resulted in the suppression of Th2-mediated responses with markedly reduced antigen-specific T-cell proliferation and eosinophil infiltration around airways and pulmonary blood vessels in a Th2-mediated allergic airway disease model (29). The differential effects of BAFF on Th1/Th2 responses may be explained by B cells. Enhanced Th1 responses have been reported to depend on B cells, while inhibition of Th2 responses by BAFF is B-cell-independent (29). Similar to these results, our data also showed that the blockade of BAFF signaling acts as a reciprocal regulator of the Th1 and Th2 populations in CD4^+^ T cells in the acute GVHD model (**Figure 3A**).

In addition, Th17 cells are a source of proinflammatory cytokines implicated in autoimmune and inflammatory conditions. Constitutive overexpression of BAFF in BAFF-Tg mice promotes Th17 cell generation *in vitro* and *in vivo* and aggravates the manifestations of Th17 cell-driven disease, experimental autoimmune encephalomyelitis, and rheumatoid arthritis (11). Induction of the Th1 and Th17 pathways predominantly suppresses Th2 and Treg cells and increases the severity of acute GVHD after allo-BMT (13). Previously, our group proposed that blockade of the IL-21 signaling pathway reduces BAFF-induced downregulation of pathogenic T- and B-cell function through the Akt–mTORC1–p70S6 kinase pathway in an acute GVHD model (9). In this study, blocking of BAFF signaling was suggested to attenuate acute GVHD through the regulation of Th1, Th17, and Treg cells, as well as B-cell homeostasis (**Figures 1**, **5**). Also, compared with the general GHVD induction mouse experimental model, the survival outcomes and acute GVHD control were increased through the effect of BAFF blockers in the GVHD mouse model purified with T cells and B cells. Based on this, BAFF may be one of the developmental mechanisms after BMT, and it could be a reasonable target to control aGVHD (**Figures 2**, **5**).

BAFF has complex and disease-dependent roles in the regulation of immune responses. For example, in transgenic mice, BAFF promoted the acceptance of islet allografts and delayed the rejection of skin grafts. The ability of BAFF to promote Treg cell expansion was not intrinsic to T cells, as Treg cells did not express high levels of BAFF-R, and excessive BAFF did not trigger the processing of NF-κB in Treg cells (30). Our group has reported that the blockade of BAFF through belimumab therapy or blocking of IL-21 signaling plays critical roles in the generation of CD4^+^CD25^+^Foxp3^+^ Treg cells in acute GVHD after allo-BMT (**Figure 3C**) (9). These observations suggest that other as yet unknown mechanisms are involved in the expansion of Treg cells by BAFF. Taken together, these results show that BAFF has dichotomous effects on T-cell immune responses.

Although our study has confirmed that the BAFF/BAFF-R pathway may have a positive role in the regulation of acute GVHD, it has limitations: our study started with the concept that the BAFF/BAFF-R pathway has an important effect on the regulation of GVHD based on previously published research (17). However, in several important BAFF-related mechanistic studies, BAFF can interact with three receptors: BAFF-R, B-cell maturation antigen (BCMA), and transmembrane activator and calcium-modulator and cyclophilin ligand interactor (TACI) on B cells (1, 31). In T cells, the transcripts of TACI were usually much higher than BAFF-R. Therefore, further research is needed on which of the BAFF/BAFF-R pathway or the BAFF/TACI pathway is more suitable for acute GVHD using BAFF-R and TACI conditional knockout mice or BAFF-R or TACI blockage antibodies. Therefore, in order to supplement and prove this content, we are currently conducting another GVHD study using BAFF-R and TACI conditional knockout mice.

In conclusion, the results of the present study show that a BAFF blockade might modulate CD4^+^ T-cell-induced acute GVHD early after allo-BMT and the possibility of simultaneously controlling chronic GVHD, which may appear later after allo-BMT.

## Data availability statement

The raw data supporting the conclusions of this article will be made available by the authors, without undue reservation.

## Ethics statement

The study protocol was approved by all institutional review boards and local ethics committees of the participating centers. All eligible patients gave written informed consent before any study-related procedure was performed. The patients/participants provided their written informed consent to participate in this study. All procedures involving animals were in accordance with the Laboratory Animals Welfare Act, the Guide for the Care and Use of Laboratory Animals, and the Guidelines and Policies for Rodent Experimentation provided by the Institutional Animal Care and Use Committee (IACUC) of the School of Medicine of the Catholic University of Korea. The study protocol was approved by the Institutional Review Board of The Catholic University of Korea (CUMC-2015-0097-02). Written informed consent was obtained from the individual(s) for the publication of any potentially identifiable images or data included in this article.

## Author contributions

S-GC designed the study. YJ, J-YL, K-IL, and NK collected the data. S-GC, YJ, J-YL, K-IL, and NK analyzed and interpreted the data. YJ, J-YL, K-IL, NK, and S-GC wrote and revised the manuscript. YJ, J-YL, K-IL, NK, and S-GC approved the final version of the manuscript. All authors contributed to the article and approved the submitted version.

## References

[1] Mackay F Figgett WA Saulep D Lepage M Hibbs ML. B-cell stage and context-dependent requirements for survival signals from BAFF and the b-cell receptor. Immunol Rev (2010) 237:205–25. doi: 10.1111/j.1600-065X.2010.00944.x 20727038

[2] Sarantopoulos S Stevenson KE Kim HT Cutler CS Bhuiya NS Schowalter M. Altered b-cell homeostasis and excess BAFF in human chronic graft-versus-host disease. Blood (2009) 113:3865–74. doi: 10.1182/blood-2008-09-177840 PMC2670799 19168788

[3] Nandi A Estess P Siegelman M. Bimolecular complex between rolling and firm adhesion receptors required for cell arrest; CD44 association with VLA-4 in T cell extravasation. Immunity (2004) 20:455–65. doi: 10.1016/S1074-7613(04)00077-9 15084274

[4] Renkonen R Hayry P. Bone marrow transplantation in the rat. i. histologic correlations and quantitation of cellular infiltrates in acute graft-versus-host disease. Am J Pathol (1984) 117:462–70. PMC1900594 6391191

[5] Leszczynski D Renkonen R Hayry P. Bone marrow transplantation in the rat. III. structure of the liver inflammatory lesion in acute graft-versus-host disease. Am J Pathol (1985) 120:316–22. PMC1887820 3895973

[6] Ringden O Witherspoon RP Storb R Ekelund E Thomas ED. Increased *in vitro* b-cell IgG secretion during acute graft-versus-host disease and infection. observations in 50 human marrow transplant recipients. Blood (1980) 55:179–86. doi: 10.1182/blood.V55.2.179.179 6986176

[7] Ratanatharathorn V Logan B Wang D Horowitz M Uberti JP Ringden O. Prior rituximab correlates with less acute graft-versus-host disease and better survival in b-cell lymphoma patients who received allogeneic peripheral blood stem cell transplantation. Br J Haematol (2009) 145:816–24. doi: 10.1111/j.1365-2141.2009.07674.x PMC3355660 19344418

[8] Chen M Lin X Liu Y Li Q Deng Y Liu Z. The function of BAFF on T helper cells in autoimmunity. Cytokine Growth Factor Rev (2014) 25:301–5. doi: 10.1016/j.cytogfr.2013.12.011 PMC4055514 24411564

[9] Lim JY Park MJ Im KI Kim N Park HS Lee SH. Interleukin 21 blockade modulates activated T- and b-cell homeostasis *via* b-cell activating factor pathway-mediated inhibition in a murine model of acute graft-versus-host disease. Exp Hematol (2015) 43:23–31.e1-2. doi: 10.1016/j.exphem.2014.09.005 25246268

[10] Ohata J Zvaifler NJ Nishio M Boyle DL Kalled SL Carson DA. Fibroblast-like synoviocytes of mesenchymal origin express functional b cell-activating factor of the TNF family in response to proinflammatory cytokines. J Immunol (2005) 174:864–70. doi: 10.4049/jimmunol.174.2.864 15634908

[11] Zhou X Xia Z Lan Q Wang J Su W Han YP. BAFF promotes Th17 cells and aggravates experimental autoimmune encephalomyelitis. PloS One (2011) 6:e23629. doi: 10.1371/journal.pone.0023629 21897850 PMC3163640

[12] Diaz GA. Released on a WHIM. Blood (2011) 118:4764–5. doi: 10.1182/blood-2011-08-375162 22053172

[13] Yu Y Wang D Liu C Kaosaard K Semple K Anasetti C. Prevention of GVHD while sparing GVL effect by targeting Th1 and Th17 transcription factor T-bet and RORgammat in mice. Blood (2011) 118:5011–20. doi: 10.1182/blood-2011-03-340315 PMC3208306 21856864

[14] Stohl W Hilbert DM. The discovery and development of belimumab: the anti-BLyS-lupus connection. Nat Biotechnol (2012) 30:69–77. doi: 10.1038/nbt.2076 22231104 PMC3264947

[15] Park JS Lim MA Cho ML Ryu JG Moon YM Jhun JY. p53 controls autoimmune arthritis *via* STAT-mediated regulation of the Th17 cell/Treg cell balance in mice. Arthritis Rheum (2013) 65:949–59. doi: 10.1002/art.37841 23280308

[16] Jia W Poe JC Su H Anand S Matsushima GK Rathmell JC. BAFF promotes heightened BCR responsiveness and manifestations of chronic GVHD after allogeneic stem cell transplantation. Blood (2021) 137:2544–57. doi: 10.1182/blood.2020008040 PMC8109011 33534893

[17] Sarantopoulos S Stevenson KE Kim HT Bhuiya NS Cutler CS Soiffer RJ. High levels of b-cell activating factor in patients with active chronic graft-versus-host disease. Clin Cancer Res (2007) 13:6107–14. doi: 10.1158/1078-0432.CCR-07-1290 PMC2941091 17947475

[18] Hu S Wang R Zhang M Liu K Tao J Tai Y. BAFF promotes T cell activation through the BAFF-BAFF-R-PI3K-Akt signaling pathway. BioMed Pharmacother (2019) 114:108796. doi: 10.1016/j.biopha.2019.108796 30921706

[19] Ng LG Sutherland AP Newton R Qian F Cachero TG Scott ML. B cell-activating factor belonging to the TNF family (BAFF)-r is the principal BAFF receptor facilitating BAFF costimulation of circulating T and b cells. J Immunol (2004) 173:807–17. doi: 10.4049/jimmunol.173.2.807 15240667

[20] Allen JL Fore MS Wooten J Roehrs PA Bhuiya NS Hoffert T. B cells from patients with chronic GVHD are activated and primed for survival *via* BAFF-mediated pathways. Blood (2012) 120:2529–36. doi: 10.1182/blood-2012-06-438911 PMC3448264 22896003

[21] Kim JS Kim SJ Cheong JW Kim Y Hwang DY Yoon S. Clinical significance of b cell-activating factor (BAFF) and a proliferation-inducing ligand (APRIL) in acute graft-versus-host disease after allogeneic hematopoietic stem cell transplantation. Korean J Hematol (2011) 46:175–9. doi: 10.5045/kjh.2011.46.3.175 PMC3208200 22065972

[22] Benitez A Torralba K Ngo M Salto LM Choi KS De Vera ME. Belimumab alters transitional b-cell subset proportions in patients with stable systemic lupus erythematosus. Lupus (2019) 28:1337–43. doi: 10.1177/0961203319869468 PMC7769209 31423896

[23] Mackay F Browning JL. BAFF: a fundamental survival factor for b cells. Nat Rev Immunol (2002) 2:465–75. doi: 10.1038/nri844 12094221

[24] Pidala J Sarwal M Roedder S Lee SJ. Biologic markers of chronic GVHD. Bone Marrow Transplant (2014) 49:324–31. doi: 10.1038/bmt.2013.97 PMC3976639 23872737

[25] Jacobson CA Sun L Kim HT McDonough SM Reynolds CG Schowalter M. Post-transplantation b cell activating factor and b cell recovery before onset of chronic graft-versus-host disease. Biol Blood Marrow Transplant (2014) 20:668–75. doi: 10.1016/j.bbmt.2014.01.021 PMC3985384 24462743

[26] Huard B Schneider P Mauri D Tschopp J French LE. T Cell costimulation by the TNF ligand BAFF. J Immunol (2001) 167:6225–31. doi: 10.4049/jimmunol.167.11.6225 11714784

[27] Scapini P Carletto A Nardelli B Calzetti F Roschke V Merigo F. Proinflammatory mediators elicit secretion of the intracellular b-lymphocyte stimulator pool (BLyS) that is stored in activated neutrophils: implications for inflammatory diseases. Blood (2005) 105:830–7. doi: 10.1182/blood-2004-02-0564 15358625

[28] Scapini P Nardelli B Nadali G Calzetti F Pizzolo G Montecucco C. G-CSF-stimulated neutrophils are a prominent source of functional BLyS. J Exp Med (2003) 197:297–302. doi: 10.1084/jem.20021343 12566413 PMC2193843

[29] Sutherland AP Ng LG Fletcher CA Shum B Newton RA Grey ST. BAFF augments certain Th1-associated inflammatory responses. J Immunol (2005) 174:5537–44. doi: 10.4049/jimmunol.174.9.5537 15843552

[30] Shiomi S Kuroki T Ueda T Ikeoka N Nishiguchi S Nakajima S. Significance of blood flow in the inferior and superior mesenteric veins for the formation of oesophageal varices. J Gastroenterol Hepatol (1991) 6:151–4. doi: 10.1111/j.1440-1746.1991.tb01456.x 1912422

[31] Vigolo M Chambers MG Willen L Chevalley D Maskos K Lammens A. A loop region of BAFF controls b cell survival and regulates recognition by different inhibitors. Nat Commun (2018) 9:1199. doi: 10.1038/s41467-018-03323-8 29572442 PMC5865128

